# Governing with public engagement: an anticipatory approach to human genome editing

**DOI:** 10.1093/scipol/scae010

**Published:** 2024-03-25

**Authors:** Dorit Barlevy, Eric Juengst, Jeffrey Kahn, Jonathan Moreno, Lauren Lambert, Alta Charo, Hervé Chneiweiss, Mahmud Farooque, David H Guston, Insoo Hyun, Paul S Knoepfler, Cynthia Selin, Rebecca Wilbanks, Manar Zaghlula, Christopher Thomas Scott

**Affiliations:** Center for Medical Ethics and Health Policy, Baylor College of Medicine, Houston, TX 77030, United States; Center for Bioethics, University of North Carolina at Chapel Hill, Chapel Hill, NC 27599, United States; Berman Institute of Bioethics, Johns Hopkins University, Baltimore, MD 21205, United States; Department of Medical Ethics and Health Policy, University of Pennsylvania, Philadelphia, PA 19104, United States; College of Global Futures, Arizona State University, Tempe, AZ 85287, United States; Law School, University of Wisconsin–Madison, Madison, WI 53706, United States; Neuroscience, Institute of Biology Paris Seine, CNRS UMR8246, INSERM U1130, Sorbonne Université, Paris 75252, France; Consortium for Science, Policy & Outcomes, Arizona State University, Washington, DC 20006, United States; School for the Future of Innovation in Society, Arizona State University, Tempe, AZ 85281, United States; School for the Future of Innovation in Society, Arizona State University, Tempe, AZ 85281, United States; Julie Ann Wrigley Global Futures Laboratory, Arizona State University, Tempe, AZ 85287, United States; Museum of Science, Boston, MA 02114, United States; Center for Bioethics, Harvard Medical School, Boston, MA 02115, United States; Department of Cell Biology & Human Anatomy, UC Davis School of Medicine, Davis, CA 95616, United States; School for the Future of Innovation in Society, Arizona State University, Tempe, AZ 85281, United States; School of Sustainability, Arizona State University, Tempe, AZ 85281, United States; University Writing Program, Johns Hopkins University, Baltimore, MD 21218, United States; Innovative Genomics Institute, University of California, Berkeley, CA 97404, United States; Center for Medical Ethics and Health Policy, Baylor College of Medicine, Houston, TX 77030, United States

**Keywords:** human genome editing, anticipatory governance, public engagement

## Abstract

In response to calls for public engagement on human genome editing (HGE), which intensified after the 2018 He Jiankui scandal that resulted in the implantation of genetically modified embryos, we detail an anticipatory approach to the governance of HGE. By soliciting multidisciplinary experts’ input on the drivers and uncertainties of HGE development, we developed a set of plausible future scenarios to ascertain publics values—specifically, their hopes and concerns regarding the novel technology and its applications. In turn, we gathered a subset of multidisciplinary experts to propose governance recommendations for HGE that incorporate identified publics’ values. These recommendations include: (1) continued participatory public engagement; (2) international harmonization and transparency of multiple governance levers such as professional and scientific societies, funders, and regulators; and (3) development of a formal whistleblower framework.

## 1. Background and introduction

In late November 2018, on the eve of the second international summit on human genome editing (HGE), news broke that Chinese biophysicist He Jiankui had successfully implanted genetically modified human embryos (Regalado 2018). Though calls for public engagement regarding HGE began before this scandal (Chneiweiss et al. 2017; Nuffield Council on Bioethics 2018), such calls intensified after it (German Ethics Council 2018; Matthews and Iltis 2019; Adashi et al. 2020; National Academy of Medicine, National Academy of Sciences, & the Royal Society 2020; Zhang, Chen, and Zhang 2021). In response to these calls, the research team (DB, LL, MF, CS, CTS) used an anticipatory approach to engage with experts and publics, in order to enable the latter to voice their hopes and concerns regarding HGE and to help inform the process by which governance policies are proposed and eventually implemented.

The anticipatory approach that informed our project draws upon a set of methods called anticipatory governance (AG). By building capacities to engage with lay-publics, integrate knowledge across disciplinary divides, and systematically explore plausible futures, AG is designed to help develop policies for the responsible research, development, and deployment of emerging technologies. AG helps to systematically explore the layers of uncertainty that arise as such technologies interface with existing and changing social and ethical norms, and it envisions an expansive set of governance mechanisms to best address prioritized areas of concern (Barben et al. 2008; Guston 2014).

Following this vision, the research team began the project by interviewing thirty experts from various disciplines who are leading authors on the science, ethics, and policy of HGE, to discuss the drivers and uncertainties of its technological development (Barlevy et al. 2023; Nelson and Selin 2023). Using this input, the research team developed a set of scenarios detailing plausible HGE futures that are grounded in the current state of technological development (Selin et al. 2023). These scenarios were then used in four public deliberation forums including a total of 150 people (three forums convened onsite in Phoenix, AZ, Boston, MA, and Waco, TX, and one convened online), to ascertain diverse American publics’ values with respect to HGE. More than half of participants were identified as female (54 per cent) and white (58 per cent), with the largest age cohort between 25-years and 44-years old (34 per cent) (Quach et al. 2022a). Many self-classified as politically liberal (46 per cent) or moderate (29 per cent) and considered faith important (31 per cent) or very important (22 per cent) (Quach et al. 2022b). The research team then thematically analyzed the data generated from these public deliberation forums (Quach et al. 2022b). The main hopes of forum participants included the prospects of targeting disease, conducting more research, implementing oversight and regulation, and increasing transparency of governance mechanisms. These hopes aligned with participants’ principal concerns over issues of accessibility, affordability, unintended effects, and rogue actors. Furthermore, forum participants were interested in using available infrastructure and resources (such as the Food and Drug Administration and institutional review boards (IRBs)) to regulate HGE, as well as involving a global oversight body. Though many forum participants desired democratic representation in policy decisions, some felt that such decisions should be made by qualified experts in various fields including ethics and law. Finally, forum participants wanted to prioritize applications of HGE to focus on targeting disease (both treatment of current disease and prevention of future disease), rare conditions, and monogenic conditions, as well as its fair and equitable distribution according to need. The research team presented these findings to a subset of the previously interviewed experts who focus specifically on HGE governance. Together, the project team and experts then collectively devised the governance proposals discussed in the section further on Governance Solutions, incorporating the values distilled from thematic analysis of the public deliberation forums. This expert workshop on the governance of HGE science and research took place against a background of social challenges over providing equitable access to its clinical fruits (Organising Committee of the Third International Summit on Human Genome Editing 2023). While these concerns depend on larger issues involving the structure of healthcare systems and efforts to achieve justice in healthcare delivery, they provide a critical context for any efforts to develop AG in this area.

This article reports on the research team’s findings and our collective reflections on these results. We first present a compressed summary of the historical context that has led to the current inflection point on HGE governance. Then we detail a set of policy recommendations, beginning with an endorsement for continued participatory public engagement and followed by suggestions on who, where, and how to best govern HGE. We conclude by acknowledging the challenges of governing this revolutionary technology and provide recommendations for overcoming geopolitical divisions to harmonize governance globally in a transparent manner.

## 2. Setting the present scene

**Figure 1.**
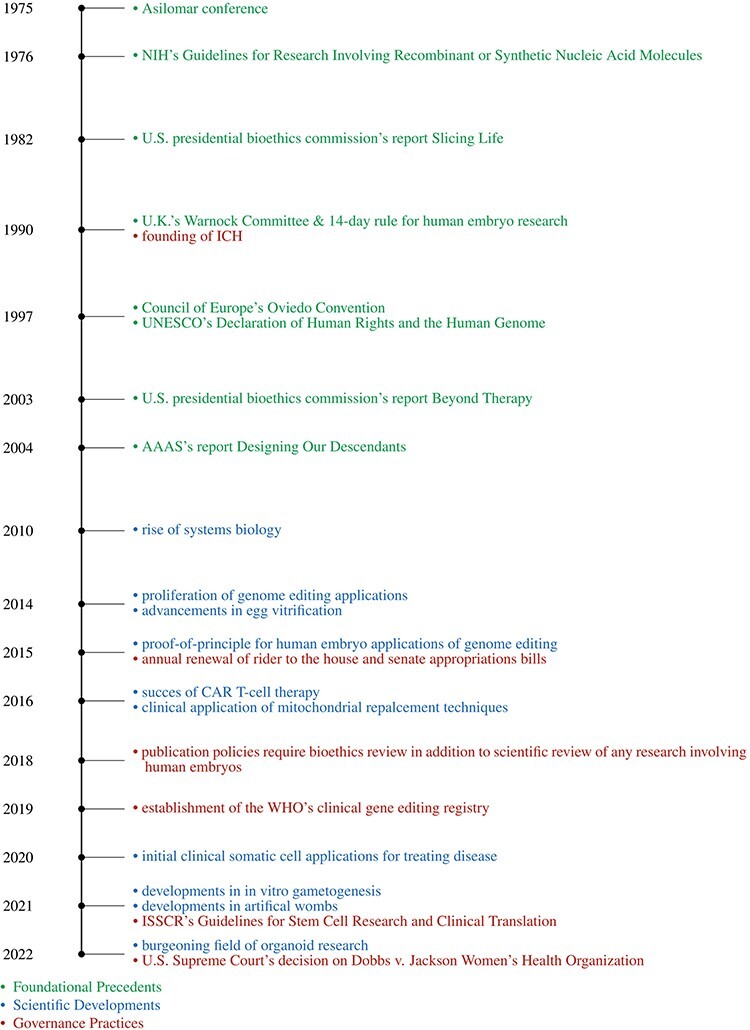
Timeline.

Before detailing our set of policy proposals for HGE, it is important to note the multiple foundational precedents, scientific developments, governance practices (see Fig. 1), and sociocultural shifts (see Table 1) that frame societies’ current opportunities for and challenges in addressing the development and application of HGE. Our AG process was embedded within this specific context, which shaped public and expert deliberations about emerging HGE research and application.

**Table 1.** Summary of sociocultural shifts.

Legacy of eugenics. Transhumanism movement. Forms of popular culture, like the movies *Gattaca* and *Elysium*. Expert disenchantment with “treatment/enhancement” as a useful boundary concept. Rise of bioethical literature defending “liberal eugenics.” Calls for broad societal consensus before embarking on controversial research. Growing excitement about biomedical citizen science (including self-experimentation and “biohacking”). Proliferation of direct-to-consumer gene testing and other forms of commercialization. Spread of misinformation and disinformation resulting in publics’ declining trust in scientific institutions. Public focus on equity due to COVID-19 pandemic and Black Lives Matter movement. Surging trend of corporate social responsibility. Shifting models of public engagement and deliberation that view publics as experts. Increasing patient demands for a libertarian “right to try” new technologies.

### 2.1 Foundational precedents

The last decade’s waves of position papers and governance reports on HGE research emerged against a much longer history of science policy initiatives relevant to human genetic modification. Paul Berg, David Baltimore, and others trace these precedents to the Asilomar Conference in 1975, partly to frame that history as a story about the scientific community’s assumption of proactive responsibility for the implications of novel scientific research (Chinese Academy of Sciences, The Royal Society, U.S. National Academy of Sciences, & U.S. National Academy of Medicine 2015). The Asilomar Conference stimulated the development of US recombinant DNA guidelines and advisory committees (Department of Health and Human Services, & National Institutes of Health 2019), which set internationally influential precedents for “proceeding with caution” in anticipating future gene transfer research. One of these precedents was the imperative to move carefully through *in vitro* and animal research before human trials, even in emergency cases of life-threatening disease. A second important precedent was the imperative to proceed transparently, with opportunities for public review and input on new human applications (Juengst and Walters 1999). Both of these foundational precedents remain influential in contemporary discussions of HGE. In the USA, these precedents were reinforced by a presidential bioethics commission’s 1982 report, *Splicing Life*, which articulated policy boundaries between somatic cell and germline gene transfer and between medical and non-medical applications of either, for both technical reasons and social policy considerations (President’s Commission for the Study of Ethical Problems in Medicine and Biomedical and Behavioral Research 1982). After two decades of experience with somatic cell gene transfer trials, these boundaries were revisited by another presidential bioethics commission’s report addressing enhancement, *Beyond Therapy* (President’s Council on Bioethics 2003) and a report from the American Association for the Advancement of Science (AAAS) reconsidering inheritable genetic interventions, *Designing Our Descendants* (Chapman and Frankel 2004). These reports helped set the stage both for contemporary philosophical skepticism about the cogency of boundaries and the search for alternative ways to articulate the ethical limits of HGE in contemporary policy reports.

Meanwhile, influential science policy developments in other countries also set important precedents for the last decade’s HGE deliberations. The UK’s Warnock Committee and its 14-day rule for human embryo research, now legally adopted in over a dozen countries (Human Fertilisation and Embryology Act 1990; Hurlbut et al. 2017), continue to provide touchstones for contemporary HGE deliberations. In addition, the Council of Europe’s Oviedo Convention, which prohibits inheritable HGE, provided important ethical frames for the discussion by citing the need to protect human rights and preserve human dignity (Council of Europe 1997). Similarly, UNESCO’s “Declaration of Human Rights and the Human Genome” advanced the idea that genomic research should be governed as the “common heritage of humankind” (UNESCO 1997). All these foundational policy precedents have echoed through public educational efforts by scientists, science popularizers, and opinion poll designers, and have informed public attitudes about these technologies (Funk, Kennedy, and Sciupac 2016; American Society of Human Genetics 2020). However, they were developed with limited, if any, sustained public engagement and therefore reflect only the knowledge and values of a relatively narrow group of experts. This leaves their lessons for contemporary governance debates, which prioritize public engagement, as imperatives for future policymaking.

### 2.2 Scientific developments

He Jiankui’s 2018 announcement of successfully implanting genome-edited embryos in women (Cyranoski and Ledford 2018) is often considered as the scientific event that galvanized recent international governance discussions of HGE research. However, numerous scientific developments paved the way for the advancement of such research and greater discussions of its governance. Some of the scientific developments that have been particularly influential in stimulating the last decade of governance discussions of HGE include those listed in Fig. 1 (e.g. proliferation of genome-editing applications, especially clustered regularly interspaced short palindromic repeats (CRISPR) due to its precision and ease of use (Gupta and Musunuru 2014); proof-of-principle for human embryo applications of genome editing (Liang et al. 2015; Kang et al. 2016; Ma et al. 2017); and the start of a first-in-human trial using CRISPR to treat sickle cell anemia (Gostimskaya 2022)). The scientific research behind almost all of these technical developments has been conducted with little, if any, anticipatory public engagement or policy development. It nevertheless provides important scientific frames for informed public deliberations about current HGE research, its public oversight, and its increasing pace. How best to communicate this science without overly privileging scientific expertise remains a challenge for designing public engagement exercises, but it is one of the challenges that our anticipatory engagement approach is intended to meet.

### 2.3 Governance practices

Although many important, country-specific policy reports on HGE have appeared over the last decade (Brokowski 2018), perhaps the most striking feature of science governance efforts that set precedents for contemporary HGE governance deliberations has been recognition of the globalization of science, the calls to globalize governance, and the need to incorporate a wider range of stakeholders into governance development, both in terms of international representation and in terms of the social sectors involved in the process. Recent expert initiatives to frame policy for HGE research have been heavily influenced by this need, even with the recognition that operationalizing this imperative across different jurisdictions and cultural attitudes toward public engagement poses significant practical and political challenges. In the past, these challenges have led to mixed governance systems involving both international consensus and local regulation, as well as soft/informal (recommendations and guidelines) and hard/formal (statutory) types of regulation, which provide additional precedents for contemporary discussions of genome editing governance (Genetic Literacy Project 2020).

Meanwhile, there have also been science policy developments that have emerged concurrently with the last decade’s HGE governance efforts and are likely to influence future deliberations on several fronts. For example, future thinking about international governance is likely to draw from the creation of the International Council for Harmonisation of Technical Requirements for Pharmaceuticals for Human Use (ICH), which strives to harmonize worldwide the development, registration, and maintenance of safe, effective, and high-quality medicines (International Council for Harmonisation of Technical Requirements for Pharmaceuticals for Human Use n.d.); establishment of the World Health Organization’s (WHO) clinical gene-editing registry (World Health Organization n.d.), which demonstrates an effort toward international collaboration in HGE governance; and the International Society for Stem Cell Research’s (ISSCR) model for broad guidelines that can be adapted according to the country-specific policies and cultural norms (International Society for Stem Cell Research 2021). Similarly, within the USA, developments like the rider to congressional appropriations, which has been renewed annually since 2015, that essentially prohibits federal funding of heritable genome-editing research (Consolidated Appropriations Act 2016) and the Supreme Court’s decision on *Dobbs v. Jackson Women’s Health Organization* (*Dobbs, State Health Officer of the Mississippi Department of Health, et al. v. Jackson Women’s Health Organization et al*. 2022) may further limit research related to heritable HGE.

### 2.4 Sociocultural shifts

Various sociocultural phenomena also frame the current challenges for public navigation of the many emerging or potential human applications of genome editing (e.g. somatic, *in utero*, or germline for therapy, prevention, enhancement, basic science, or military use). Despite continued public opposition to enhancement and germline applications (Funk, Kennedy, and Sciupac 2016; Funk and Hefferon 2018), in the last two decades conceptual and ethical critiques have begun to put pressure on these policy barriers. In the bioethics literature, these critiques were sometimes paired with positive arguments for “liberal eugenics” and “transhumanism” (Agar 2008; Sorgner 2018). These academic developments swim upstream against public fears of a resurgence of the historical eugenics movement (Turda 2022) and the societal impact of the cautionary messages of popular culture, like the movies *Gattaca* and *Elysium* (Greenbaum and Gerstein 2022). Nevertheless, academic critiques of the traditional policy boundaries (Bostrom and Roache 2008; Rulli 2019; McGee 2020) have set precedents that open the doors to both enhancement and germline applications in contemporary deliberations of HGE governance (National Academy of Medicine, National Academy of Sciences, & the Royal Society 2020; WHO Expert Advisory Committee on Developing Global Standards for Governance and Oversight of Human Genome Editing 2021). These expert shifts paralleled the rise of commercialization in science and creation of markets for non-medical uses of genome editing (Allyse et al. 2018), patients’ demands for a libertarian “right to try” new technologies despite their risks (U.S. Food and Drug Administration 2022), and growing excitement about biomedical citizen science (including self-experimentation and “biohacking”) (Pauwels 2018; Trejo et al. 2020). At the same time, an emerging social focus on equity issues (Blankenship and Reeves 2020; Ford, Reber, and Reeves 2020; Reeves and Rothwell 2020), increasing private sector interest in corporate social responsibility (Dashwood 2020), declining public trust in science (Iyengar and Massey 2019), growing calls for broad societal consensus before embarking on controversial research (Organizing Committee for the International Summit on Human Gene Editing 2015), the emergence of non-government organizations focused on HGE (e.g. the Association for Responsible Research and Innovation in Genome Editing (ARRIGE) and the Global Observatory for Genome Editing), and social support models of public engagement that view publics as experts (Scheinerman 2023) are also becoming increasingly important, reintroducing public concerns about the potential long-term social impacts of genome-editing technologies into the governance debates.

## 3. Governance solutions

Using methods in AG, we reflected upon the project’s public deliberations and arrived at recommendations for HGE governance.^1^ We group these recommendations into the following domains: (1) robust public engagement; (2) who should govern; (3) the sites of governance; and (4) how to conduct responsible governance. This final category we separate into the development of professional norms and reporting (whistleblowing) with the understanding that no normative framework can always prevent occasional instances of irresponsible research. (See Table 2 for a summary of these recommendations.)

**Table 2.** HGE governance recommendations.

**1—Robust public engagement** To be conducted iteratively as future-based exercises. Focused on eliciting public values. Facilitation via science museums, associations, and professional societies. Giving publics the opportunity to shape the direction of the technology. Incorporating non-partisan social media. Fighting misinformation while using civil discourse.
**2—Multiple governance levers** Continued engagement with science communities and publics across boundaries of geopolitical divide. Inclusion of voices outside the boundaries of self-regulation in professional societies’ policy positions and recommendations for practice. Promotion of responsible research via an international consortium of funders, granting of intellectual property rights, and guidelines for practice and publication. International professional societies develop and disseminate guidelines that require public engagement deliberations and are adopted by other organizations with influence over researchers and institutions.
**3—Harmonization** Transnational governance to be debated and refined through coordinated public engagement. Regulatory agencies in nations active in HGE research should coordinate efforts in: – Evaluation of scientific and preclinical evidence to recommend course of action for first-in-human clinical trials. – — Establishment of priorities to commence and continue lines of basic and preclinical research in target diseases with the highest possibility of scientific and clinical success. – — Conduct of clinical trials where there is sufficient unmet medical need. – — Recommendation and launch of trials in jurisdictions that need them the most.
**4—Formal whistleblower framework** WHO-sponsored reporting agency receives anonymous “expressions of concern” and coordinates with national bodies and societies, which can take investigative steps and punitive actions.

### 3.1 Robust public engagement

National and international policy recommendations have uniformly called for public engagement prior to determining directions for and oversight of HGE (Nuffield Council on Bioethics 2016, 2018; National Academies of Sciences Engineering and Medicine 2017; German Ethics Council 2018). Though consistent across policy documents, such calls offer little detail about how most effectively to engage the public. “Engagement” in biomedical policymaking has a wide rubric, from one-way public comment periods to deeper deliberations that seek to uncover the values and beliefs of those who might use and benefit from the technologies, or be harmed by them. While there are various US federal requirements for public comment on proposed regulations, historically, these have been inconsistently applied and lack the rigor of validated engagement methods. Moreover, passive, one-way public comment is one of the least robust forms of public engagement. One model identified as an exemplar of public engagement is France’s National Ethics Committee (CCNE), which since 2011 has been charged with employing surveys, questionnaires, public debates, hearings, and a citizen’s jury to render opinions on genetic technologies to the French parliament. French law states that “any reform project on the ethical problems and social issues raised by advances in knowledge in the fields of biology, medicine, and health must be preceded by a public debate” (LOI relative à la bioéthique 2011). Another exemplar is the UK’s Nuffield Council’s use of surveys, focus groups, and interviews to advise parliamentary policy surrounding mitochondrial replacement therapy (Finnegan 2012). A third, and perhaps best-known, example is the Warnock Committee, which was the first group in the UK to consider the ethical, legal, and social implications of the science of human fertilization and embryology. Its report, which over the course of 6 years of public consultation and submission, included 695 opinions from the public in addition to 300 organizations and individuals working in reproductive sciences (Hurlbut et al. 2017).

Many commissions, reports, and scholars have argued that public engagement must be a prerequisite of policymaking. Robust governance initiatives can conduct public deliberations iteratively as future-based exercises, which can be useful for fast-moving fields such as HGE. Public deliberation forums should be alternatives to expert-driven models designed to address a “knowledge deficit” with non-scientist publics and instead focus on publics’ visions of HGE applications (Reincke, Bredenoord, and van Mil 2020). The goal should be engagement methods that focus on eliciting public values rather than scientific details.

Our engagements were conducted through the Expert and Citizen Assessment of Science and Technology (ECAST) network of academic, informal science education, and policy research organizations led by the Consortium of Science, Policy and Outcomes at Arizona State University and the Museum of Science, Boston. ECAST’s participatory technology assessment (pTA) methodology uses expert and citizen framing to inform deliberative public forums at museums to inform policy and decision-making and create broader societal engagement (Sclove 2010; Kaplan et al. 2021). Indeed, an international array of science museums could be a fertile place where engagement could proceed. There may be other suitable sites such as the AAAS, the European Union’s (EU’s) EuroScience, or professional organizations such as the American and European societies for human genetics or gene and cell therapies. Furthermore, the Global Citizen Assembly on Genome Editing is an example of scaling public deliberations from a national to global scope (Dryzek et al. 2020).

Whatever form engagement takes, the idea is not only to provide essential information (and avoid information overload) but also to give publics the opportunity to participate in shaping the direction of the technology by actively participating in its governance (Gutmann and Wagner 2017). In the USA, we contend there is a danger of HGE becoming further politicized (as embryonic stem cell research was before it). Additionally, social media currently has an outsized impact on public discourse, and thus it is important to find platforms that are not vulnerable to being flooded with repetitive and extraneous comments. Furthermore, there are clear advantages to deliberative exercises with the public as a way to fight misinformation and bridge the “divide” between experts and publics, while using civil discourse to establish responsible science policy.

### 3.2 Who should govern?

Participants in the project’s public deliberative forums articulated the view that the general public as well as experts (in science and ethics), individuals who might be recipients of HGE, and government agencies should be involved in HGE governance. They also held that representation of these stakeholders be democratic and interdisciplinary. The most salient theme from the forums was that a new global governing body should be formed by nations researching and practicing HGE.

What forms of governance might embody these principles? Though international relations have been organized by a rules-based order developed by the USA and its allies following World War II, the elements of hegemonic power granted under this system are under stress. The Russian invasion of Ukraine is widely considered to be a profound threat to this postwar order; President Putin himself has said that his goal is to obliterate the system that, in his view, has served “Anglo Saxon” interests to the detriment of the rest of the world (Antonova and Lagutina 2023). Despite these stresses, the rules-based order provides a framework for professional self-regulation, as exemplified by the successive versions of the Declaration of Helsinki (World Medical Association 2013).

In the project’s public deliberations, participants voiced concern over the potential of rogue actors using HGE for unethical purposes, and our collective ability to prevent future controversies like that of He Jiankui. In the weeks following the announcement of his HGE experiment, some observers wondered if this would not be an opportunity for China to go its own way. However, the voices of scientists in China aligned with those of the global medical science community in condemning the experiment and sanctions against He and two of his collaborators were substantial; indeed, imprisonment exceeded what could plausibly be expected in Western countries for similar actions.

Using these values uncovered from the public deliberation forums, we invoked the “invisible college,” which has been used to describe the seventeenth century correspondence among scholars that included Robert Boyle and the Royal Society who gathered for the pursuit of the public good (Kassell 2010). One interpretation of the 2018 He Jiankui episode is that the Chinese political system decided to yield to the invisible college of the international community of life scientists, one in which China’s scientists are deeply integrated. A second instance of the invisible college’s norms at work was the immediate outcry following the announcement by a Russian researcher of his intention to deploy germline editing in the same gene, *CCR5*, allegedly with less risk to offspring (Cyranoski 2019), and his eventual withdrawal of the experiment. We raise these examples of how a web of intellectual and personal connections, with broad engagement from the public could be sustained and strengthened to build soft governance capacity for the future of HGE, especially when political tensions among some nations are particularly strained. In times of international political tensions or pandemics, it is especially important to continue engaging with science communities and publics across boundaries of geopolitical divide. For example, at the height of the Cold War and the polio epidemic, the USA arranged for the dissemination of the Sabin vaccine in the Soviet Union and Eastern European states. Furthermore, the COVID-19 pandemic showed the increasing risks that accumulate when social bonds between China and the USA deteriorate (Christensen 2020).

Professional societies emerge when the invisible college becomes organized around scientific affinities and interests. There are many relevant scientific societies that have published policy positions and recommendations on the practice of HGE (Ormond et al. 2017; European Society of Gene and Cell Therapy, British Society for Gene and Cell Therapy, Deutsche Gesellschaft für Gentherapie, Finnish Society of Gene Therapy, Hellenic Society of Gene Therapy and Regenerative Medicine, Netherlands Society for Gene & Cell Therapy, Sociedad Española de Terapia Génica y Celular et al. 2018; Lovell-Badge et al. 2021; National Society of Genetic Counselors 2023), and in one case an attempt was made to harmonize normative statements among nine international organizations on issues such as germline editing (Brokowski 2018). We note, however, that while guidelines and oaths can in principle set broad outlines for responsible conduct of research, they lack the clout of regulation and law. In sum, the project’s conversations with the public revealed a basic trust in experts to govern HGE, but emphasized broader representation to ensure that voices outside the boundaries of self-regulation are heard and considered.

### 3.3 Sites of governance

The project’s public forum participants raised the need for a new, pluralistic global entity that should not be dominated by a single interest or actor. Despite relative agreement among groups about a desire for HGE research to be conducted in the interests of the public good, where the entity should reside (e.g. a scientific or research institution, the government, or private industry), and which stakeholders should therefore preside within it, was a matter of debate.

It is clear that responsible governance of HGE will not be a monolithic undertaking, especially considering the various histories and norms of nations participating in human genetic research generally. Levers of governance can be constructed in many ways and can include policy, regulation, law, professional norms, codes, rules, and societal influences, such as religious beliefs, historical legacies, education, and political conventions (Ostrom 2015). Models could be centrally focused or highly distributed among local jurisdictions and organizations (Marchant 2021). Loci of governance may include professional societies, funders, individual institutions, private actors such as industry, and global bodies such as the WHO, alongside traditional mechanisms of laws and regulation. We maintain that robust levels of participatory public engagement with a forward-looking focus should be reliably applied to any level of governance.

As other examples of governance (bans, moratoria, funding restrictions) may lose their relevance in a globalized and diffuse geopolitical order, a combination of levers may be most effective in governing HGE in the longer-term future (Marchant 2021). If governance is rooted in a shared understanding of science, then it should include the public’s understanding of science as it is delivered through outside channels, such as formal and informal science education, citizen science, and science communication through the media (Hurlbut 2015).

In our discussion of publics’ values regarding HGE (as synthesized from the project’s public deliberations data), the WHO emerged as a possible international locus for reporting deviations from norms for responsible research (see Section 3.4.3 further). Other models include an international consortium of funders, which would agree to a set of norms that scientists and clinicians would follow in order for projects and trials to be considered for grants and other means of support. A more pragmatic approach could include a distributed network of governance, with levers of control existing at many different catchpoints in the innovation process. Such a scheme could also include socially responsible licensing of intellectual property (Guerrini et al. 2017), conditions of funding, publishing guidelines with ethics and peer oversight, and professional norms through societies.

In terms of governance that would promulgate guidelines, we suggest that a professional society with an international sweep, such as the ISSCR or the International Society of Gene and Cell Therapy (ISGCT), serve in this role. Such a society or consortium of them could, with robust public engagement efforts, develop and promulgate guidelines that might be adopted by other organizations with influence over researchers and institutions, such as funders, publishers, state agencies, foundations, and trusts. ISSCR’s rules for stem cell research and clinical translation are one example of guidelines that are widely referenced and used (Lovell-Badge et al. 2021). Public engagement at the society level could follow an anticipatory approach that uses pTA methodology and a distributed network of museums, as our model did. The National Academy of Sciences, Engineering, and Medicine’s consensus committees, for example, could benefit greatly from participatory public engagement as part of their expert deliberations, fulfilling their own recommendation that public engagement is a critical need (National Academies of Sciences Engineering and Medicine 2017; National Academy of Medicine, National Academy of Sciences, & the Royal Society 2020).

### 3.4 How to govern?

We propose two complementary governance mechanisms that can be brought to bear on HGE research. First is an international governance framework that is harmonized, transparent, generalizable, and adaptive. Second is a process where HGE actors (including institutions and nations) can be held accountable for irresponsible research. These are long-term solutions, as they will require a measure of international consensus and enforcement.

#### 3.4.1 Harmonization.

One achievement of the post-World War II order has been harmonization of various governance sectors among sovereign states. Examples of harmonization range from finance and banking to intellectual property regimes and travel documents to establish identity (Farrell and Saloner 1985; Higgins and Hallstrom 2007). Technical standards for the evaluation and production of new medicines is another example, though one that has been shaken by the pandemic emergency regarding COVID-19 vaccines (Knezevic et al. 2022). In other areas, such as the rights of immigrants and refugees, the situation has deteriorated (Aspinall and Watters 2010; Borrett et al. 2019). Moreover, in some others, like the regulation of nuclear weapons production, harmonization has never been fully achieved (Joyner 2011). In the case of genetic science, the United Nations (UN) has developed documents (e.g. the Universal Declaration on the Human Genome and Human Rights) (UNESCO 1997) and institutional structures (e.g. the International Bioethics Committee and the Intergovernmental Bioethics Committee) (Bagheri, Moreno and Semplici 2015) as well as various reports and recommendations of several science academies, though these lack the force of international law. Harmonization should be attempted with the proviso that unanimity among nations in developing universal governance regimes is likely an unattainable goal, but that nations conducting HGE might come together to play different (and additive) orchestral parts. An example is the UK’s Human Fertilization and Embryology Authority’s (HFEA) licensure for human embryo research. If the agency is very concerned about how a laboratory is performing, it can suspend the license, or in extreme circumstances, revoke it (Human Fertilisation & Embryology Authority 2024). Additionally, the rationale for transnational governance should be debated and refined through coordinated participatory public engagement.

In order to achieve a working harmonization, there would need to be general agreement on the composition and remit of an international body composed of representatives from countries active in or possible beneficiaries of HGE research. This body could deliberate on three interrelated tasks: (1) through coordination of national regulatory agencies, transparently evaluate the totality of scientific and pre-clinical evidence to chart a path for first-in-human clinical trials, should the data be deemed sufficient to proceed; (2) with this evidence, prioritize commencing and continuing lines of basic and pre-clinical research in target diseases where a comprehensive risk/benefit analysis reveals the possibility of scientific and clinical success is high; and (3) conduct clinical trials for therapies with sufficient unmet medical need as well as in jurisdictions that need them the most. Some of these steps have been echoed by international bodies (Adashi and Cohen 2020; WHO Expert Advisory Committee on Developing Global Standards for Governance and Oversight of Human Genome Editing 2021). These steps align strongly with the values uncovered in our deliberations with publics, which include proceeding cautiously but deliberately with sufficient scientific evidence and in ways that take into consideration health disparities and notions of distributive justice, addressing informed expressions of public concern, and generally calling for transparency among communities of science and governments to share data and information for the benefit of all.

#### 3.4.2 Transparency for translation.

Any coordination should include agreement on the steps in translational pathways using HGE (Institute of Medicine 2014). We propose that a consortium of regulatory agencies in countries responsible for the potential use of HGE technologies require sponsors of these first-in-human trials to coordinate and share data (just as they do now with multisite, multinational pharmaceutical studies) and develop testing milestones to speed approvals for safe, efficacious treatments. Because of disparate norms and values for participating countries, we believe it is essential to develop priorities for science and translational medicine with robust and repeated efforts to engage publics. This will require agreed upon decision points and approvals across national agencies, with greater flexibility for agencies to fast-track approvals made in other countries.

A multinational coordinating agency prioritizing translational pathways is not without its challenges. Confidentiality of data would have to be waived by member states and corporations developing treatments. Other value-laden decisions could include valuations of unmet medical need, balancing the chance of an occasional adverse event against wide-spread but perhaps incremental benefits, and resource constraints in countries where there are significant health disparities. In addition, public health goals may not be consistent with where the scientific opportunities might be greatest, such as the feasibility of targeting rare monogenic disease versus the intractability of widespread multifactorial illness. Our engagement data and interviews show a wide variety of opinions about which diseases or applications should take priority, including orphan diseases and somatic editing.

As described earlier, an international consortium of funders could serve as one governance lever, especially for first-in-human trials using germline applications. Funders could adopt, expand, and implement harmonized guidelines promulgated by international agencies like WHO or organizations such as ISSCR. Funding would be contingent upon promises to abide by these rules of conduct, which could in turn intersect with similar rules adopted by peer-reviewed journals as a condition of publication. Our research interviewing thirty HGE experts revealed that peer review of socially controversial work could function as a means of governance, especially when connected to standardized global guidelines and augmented by bioethics expertise and public input. For instance, if He Jiankui had known early on that his unethical experiments would have been rejected at scientific journals, he may not have attempted them in the first place (Sharma and Scott 2015). A successful example of publishing rules can be found in the 2005 consensus by the International Committee of Medical Journal Editors, which requires investigators to deposit clinical trials information in accredited public registries before considering manuscripts for review (Zarin et al. 2017).

#### 3.4.3 Independent whistleblower.

In our study, we used the backdrop of the He Jiankui scandal to examine questions of “who should govern?” and “who is responsible?” We found unifying themes about governance among experts and publics, such as the involvement of scientists and ethicists. Our participatory public engagement exercises underscore the fact that citizens expect scientists to help oversee HGE research and to do it responsibly. With our publics, we examined the controversy surrounding He Jiankui, which prompted the Nobel laureate David Baltimore to remark at the Hong Kong summit when news of the procedure was announced that it was “a failure of self-regulation by the scientific community.” Baltimore further remarked that “we had no authority to stop him,” which “is the dilemma in trying to police the international scientific world” (Wee 2019). A hallmark of the controversy was the wide range of international actors involved, including academic collaborators, mentors, company executives, a Nobel Prize winner, and dozens of other individuals who knew or suspected what He Jiankui was doing before it became public—his so-called “circle of trust” (Cohen 2019).

Regarding the responsible governance of HGE, one scholar says, “Science should think hard about encouraging, or even requiring, scientists to inform someone of their concerns about on-going research” (Greely 2019). But what are these obligations? To report illegal or unethical research, dangerous or frivolous work? What should a scientist do about hearsay of unethical behavior? Where is the locus of the reporting function? What actions should be taken once irresponsible research is revealed? With the notion of a WHO-sponsored reporting agency as a first step, governance for irresponsible research could proceed as a place where “expressions of concern” can be fielded from scientists and then follow a pathway outlined above. We reject notions of control through punitive means exercised by organizations such as the FBI, a suggestion raised in separate interviews we conducted with individuals connected to the He Jiankui scandal; these are crime-driven organizations and some research misconduct may not actually be a crime (either because no law exists to be broken or because norms are idiosyncratic and often professionally driven).

A global version of the US Office of Research Integrity might be one solution, developed by a consortium of major funders (e.g. National Science Foundation, National Institutes of Health (NIH), Wellcome Trust, National Institute for Health and Care Research, Medical Research Council, European Research Council, Japan Agency for Medical Research and Development, Swedish Research Council). This solution could include appointed “mandatory reporters” in institutions that receive HGE funding for human translational research, based on federal rules and regulations made through the office. Major “trigger” actions (such as germline editing with the intent to implant) would be listed. A “handoff” solution may be another governance option. In this scenario, the WHO receives “expressions of concern” and then coordinates with national bodies and societies, which would broker these cases to agencies (funders, legislative groups, watchdogs) that could enforce any sanctions or actions.

The drawbacks of a formal whistleblower framework are that it: (1) might be abused or become chaotic, with people telling on everyone else (vigilantism); (2) may become overly cumbersome, as one must have a formal process for investigation; (3) requires establishing methods for protecting the whistleblower from retaliation (Lubalin and Matheson 1999); (4) can be hindered by the reluctance of some individuals to come forward out of reputational concern; (5) necessitates some sort of final adjudication; and (6) lacks an obvious mechanism for addressing public expressions of concern. A reporting framework might be constructed to be internal to the various institutions conducting HGE or external, such as a professional society, an organization such as WHO, or more familiarly, the media. Whatever model is chosen must account for cultural and value differences in the jurisdictions which participate (Nayir and Herzig 2012). There is the added complication that mechanisms do not exist for whistleblowing on countries that might permit the irresponsible research of their scientists by failing to act or enforce rules of conduct. Internal reporting systems might also suffer from professionals unwilling to testify against each other, as exemplified in malpractice and reporting medical errors (Mohammadi et al. 2019).

Our consensus view is that a form of “soft” governance with anonymous “expressions of concern” being forwarded to an international body such as WHO, which then hands threshold cases to national bodies that can take investigative steps and punitive actions is a plausible long-term strategy for whistleblower governance.

## 4. Conclusion

In sum, we see an interlocking series of governing levers for the practice of HGE that are both internationally- and nationally-based. Before considering any governance framework, policymakers in different jurisdictions should robustly and repeatedly engage their publics and do so in ways that anticipate the future directions the technology might take. Once public values reflecting the cultural norms of different jurisdictions are identified, the task becomes to develop and promulgate an overarching, harmonized framework of governance norms that is general enough to encompass a multinational set of values-based rules (informed by public concerns), yet specific enough to promote the responsible conduct of HGE research across different jurisdictions by (1) setting conditions on the approval, funding, and publishing of HGE research, and (2) constructing a pathway of reporting and oversight for instances of suspected irresponsible research that may have occurred outside the normative frameworks of conduct.

Achieving harmonization and transparency in a governance framework has challenges. There can be breakdowns even in an adherence to a rules-based order. Having an IRB in Thailand for the same clinical trials as an IRB in France or an IRB in the USA, for example, displays not only an instance of fragmentation within the rules-based order of drug and device development, but also adherence to a basic norm of independent prior review. If science policy is a product of a rules-based order, continuing to harmonize across increasingly fragmented geopolitical divides will be a central challenge in governing HGE. Efforts to globalize genetics governance will be most successful if they focus on multiple and overlapping scales and across multiple communities. These communities ought to directly not only include the “usual suspects” of the WHO, the UN, and the EU, but also ought to include multilayered public engagement and scientific research communities in various jurisdictions. In light of values underscored by our publics’ deliberation data, we recommend that the normative framework for such an effort be developed by a consortium of national, international, and professional organizations led by WHO, including the Chinese Academy of Sciences, France’s ARRIGE, and the EU’s European Group on Ethics, in concert with professional societies with practicing members of HGE (ISSCR, ISGCT, the American Society of Human Genetics, and others). The principles, once ratified, would be adopted by funding agencies, local and national jurisdictions, institutions, and publishers as thresholds for HGE researchers and practitioners who require funds, approvals, or publications necessary for the advancement of their work. Reports of irresponsible research would flow first to WHO then back to the named professional societies, which would review and pass such reports to the funders and oversight bodies therein, and local or jurisdictional regulators for adjudication.

**Figure 2.**
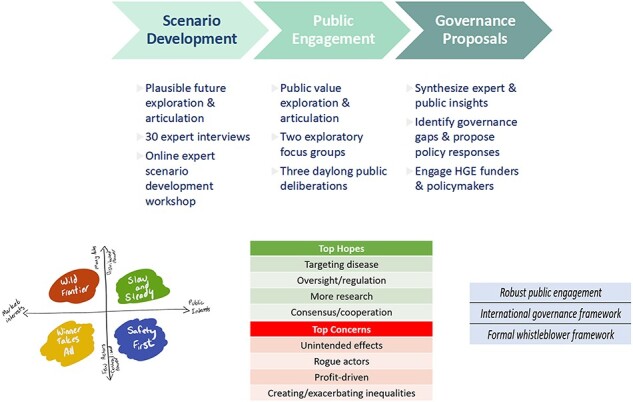
Anticipatory governance for HGE.

Our project was the first NIH-funded ELSI (Ethical, Legal and Social Implications) research to successfully deploy a full cycle of AG to HGE technologies. We stress that our methods to robustly engage publics (as described earlier and illustrated in Fig. 2) can be used in smaller-scale governance efforts (such as priorities for a funding agency) as well as national and international imperatives that seek to provide policy frameworks that are forward-looking and flexible.

## Data Availability

Workbook data from our publics deliberation forums will be made available upon reasonable request to the corresponding author.

## Footnotes

1. Though the ethical and technical implications of HGE can vary depending on the type of cells being targeted (e.g. somatic versus germline), these recommendations are not application-specific, reflecting public deliberation participants’ diverse opinions with respect to the acceptability of germline editing.
